# Intra-Peritoneal Rectal Perforation in a Neonate Leading to Acquired Rectal Atresia

**Published:** 2013-04-01

**Authors:** Yogesh Kumar Sarin

**Affiliations:** Department of Pediatric Surgery, Maulana Azad Medical College, New Delhi-110002, INDIA

**Keywords:** Neonate, Rectal perforation, Acquired rectal atresia

## Abstract

A neonate sustaining iatrogenic rectal injury that led to acquired rectal atresia is reported. It was successfully treated by transanal, end-to-end, rectorectal anastomosis.

## INTRODUCTION

Pneumoperitoneum in a newborn is commonly caused by necrotising enterocolitis or spontaneous gastric perforation. Occasionally, pneumoperitoneum has been reported in neonatal units due to iatrogenic rectal injuries [1]. Although considerable morbidity and high mortality is known to occur after iatrogenic colorectal injuries in neonates [2], an acquired rectal atresia following a rectal injury has not been reported hitherto. Such a complication in a neglected case of neonatal rectal perforation is reported.

## CASE REPORT

A 4-day-old, 1.7kg weight male neonate, born at term to a primigravida mother by normal vaginal delivery conducted by an untrained birth attendant, was brought with complaints of abdominal distension from D2 of life. There was no antenatal checkup or tetanus immunization, although the pregnancy and delivery was reported as normal. Although he had passed meconium on D1 of life, he had not passed any meconium for the previous three days. On examination, he was sick looking and had tachycardia (PR=160/min.) and tachypneic (RR=60/min.). Abdomen was grossly distended and tense. Hematological and biochemical investigations were normal. Abdominal X-ray showed massive pneumoperitoneum (Fig. 1). A clinical diagnosis of necrotizing enterocolitis Bell’s stage III was made. Keeping in view of the respiratory distress resulting from massive pneumoperitoneum, bilateral peritoneal drains were immediately put to deflate the abdomen. 

On exploration few hours later, there was gross contamination of peritoneal cavity with meconium. There was a rent in anterior wall of rectum reaching up to the peritoneal reflection; rest of the gut was normal. During surgery, a pack of cotton wool pack was found in the rectal lumen. Congenital rectal atresia was ruled out by passing a Hegar dilator from below. A proximal diverting colostomy at the level of sigmoid colon was done. Multiple sero-muscular colonic biopsies were taken just proximal and distal to this colostomy. No attempt was made to repair the rectal perforation.

The postoperative period was uneventful. The colonic biopsies ruled out Hirschsprung’s disease. The relatives denied any knowledge about how rectal perforation could have occurred or who left the cotton wool pack in the rectum.

The child was lost to follow up for a year. Per rectal examination revealed that rectum was totally obstructed about 4cm from anal verge. Distal cologram showed complete obstruction of rectum. The rectal obstruction was only few millimeters thick and simulated like congenital rectal atresia (Fig. 1). 

**Figure:**
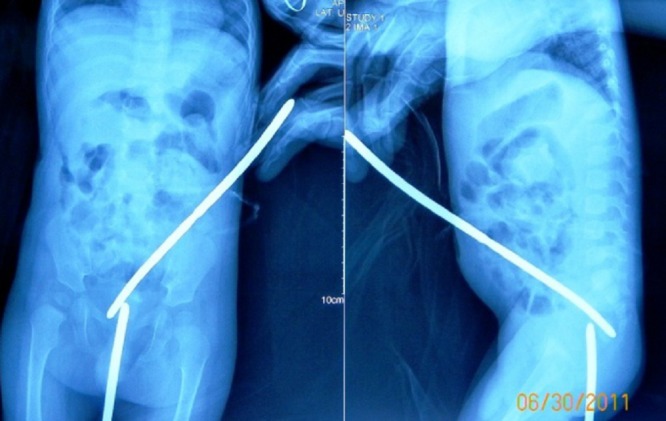
Figure 1: Plain roentgenograms with bougies inserted from sigmoid colostomy and anal opening, revealing a thin membranous rectal atresia.

Transanal, end-to-end, rectorectal anastomosis (TERA) was done for the membranous acquired rectal atresia. The passage created was dilated over next six weeks and this was followed by closure of colostomy. Both the surgeries went uneventfully and the child has been doing well on two year follow up.

## DISCUSSION

Spontaneous rectal perforation in a neonate not born with anorectal malformation is unknown. However, iatrogenic perforations of the rectosigmoid are frequent [2]. Rectal perforations in neonates caused by incorrect use of rectal cannula [3], Hegar dilator [4], thermometer [1, 5] etc. have been reported. They have been also reported due to therapeutic saline enemas [6] and diagnostic barium enemas [7]. The exact cause of rectal perforation in the index case is not known, but finding of cotton wool within rectal lumen close to rectal perforation suggests possible finger stimulation by the untrained birth attendant. 

Thermometer-induced rectal perforation is obviously caused by wrong technique of insertion of thermometer [1, 8]. Nurses should be instructed to insert the rectal thermometer into the anal canal and then advance at an angle of 30° backwards, not straight into the rectum parallel to the cot as one so often sees [8]. The suggested mechanisms of injury by enemas include trauma from tip of enema tube, overinflation of the balloon, in case a Foley’s catheter is used and increased hydrostatic pressure [7].

It is easy to diagnose intraperitoneal rectal perforation resulting in pneumoperitoneum, but extraperitoneal rectal perforations without perineal wound or pelvic fracture are difficult to diagnose and are often missed [9]. The diagnostic difficulty in case of extraperitoneal rectal perforation in neonates is enhanced as we usually avoid doing a digital rectal examination or proctosigmoidoscopy on them. The extraperitoneal rectal perforation may eventually lead to infection in the presacral space, retroperitoneal space and/or lead to ischiorectal and pelvirectal abscess. A minor extra-peritoneal rectal perforation may be treated non-operatively with antibiotics, while intra-peritoneal perforation causing pneumoperitoneum such as in index case would obviously need operative intervention. Significant morbidity and mortality has been known to occur in case there is a delay in seeking treatment [9]. 

The highlight of this index case is the occurrence of acquired rectal atresia. Acquired rectal atresias are known to occur after reoperations in anorectal malformations [10]; these are most likely related to tension related to inadequate mobilization, or rectal devascularization/ ischemia. A similar devascularisation/ischemia pathogenesis is suggested for this case too. 

Various operative approaches such as abdomino-perineal, sacro-perineal, Duhamel’s procedure, Stephen’s posterior approach, Soave pull through, posterior sagittal approach, TERA, transanal endorectal pull through (TERPT), single stage sacral approach, abdominal and transanal approach, single transanal approach, laparoscopic and transanal approach, etc. have been used for the correction of both congenital and acquired rectal atresias [11]. Each surgical technique has its own advantages and disadvantages. We performed TERA in our patient. 

Upadhyaya [12] was first to describe TERA technique for the surgical correction of rectal atresia. The rationale of TERA is based on three factors: (1) the anorectal canal distal to the atresia is normally developed, as are the sphincteric muscles surrounding it; (2) the anorectum can be preoperatively dilated to allow a transanal anastomosis of good size; and (3) the atretic segment can be effectively "intussuscepted" into the anal canal, almost up to the anal verge, by an oversized metal bougie passed through the sigmoid colostomy. A midline sagittal incision over the metal bougie exposes the rectal pouch, which is mobilized from the surrounding muscle fibers, and a direct, end-to-end anastomosis is performed. Upadhyaya felt that performing abdominoperineal or sacroperineal procedures entailed major traumatizing surgery with an inherent risk of complications. Unlike posterior sagittal approach, no sphincters need to be divided, thus better outcome as regards to bowel continence are expected.

## Footnotes

**Source of Support:** Nil

**Conflict of Interest:** The author belongs to the editorial team, however the manuscript is dealt independently by other editors and the author did not participate in decision making of the manuscript.
